# Degron tagging for rapid protein degradation in mice

**DOI:** 10.1242/dmm.050613

**Published:** 2024-04-26

**Authors:** Brianda A. Hernández-Morán, Gillian Taylor, Álvaro Lorente-Macías, Andrew J. Wood

**Affiliations:** ^1^ MRC Human Genetics Unit, Institute of Genetics and Cancer, University of Edinburgh, Crewe Road, Edinburgh EH4, 2XR, UK; ^2^ Edinburgh Cancer Research, Cancer Research UK Scotland Centre, Institute of Genetics and Cancer, University of Edinburgh, Crewe Road, Edinburgh EH4 2XR, UK

**Keywords:** Protein degradation, Target validation, Mouse models

## Abstract

Degron tagging allows proteins of interest to be rapidly degraded, in a reversible and tuneable manner, in response to a chemical stimulus. This provides numerous opportunities for understanding disease mechanisms, modelling therapeutic interventions and constructing synthetic gene networks. In recent years, many laboratories have applied degron tagging successfully in cultured mammalian cells, spurred by rapid advances in the fields of genome editing and targeted protein degradation. In this At a Glance article, we focus on recent efforts to apply degron tagging in mouse models, discussing the distinct set of challenges and opportunities posed by the *in vivo* environment.

## Teaser

**Summary:** We review recent applications of degron tagging in mouse models, focusing on the challenges and opportunities for using this technology to rapidly degrade proteins of interest in mammalian tissues.

**Figure:**
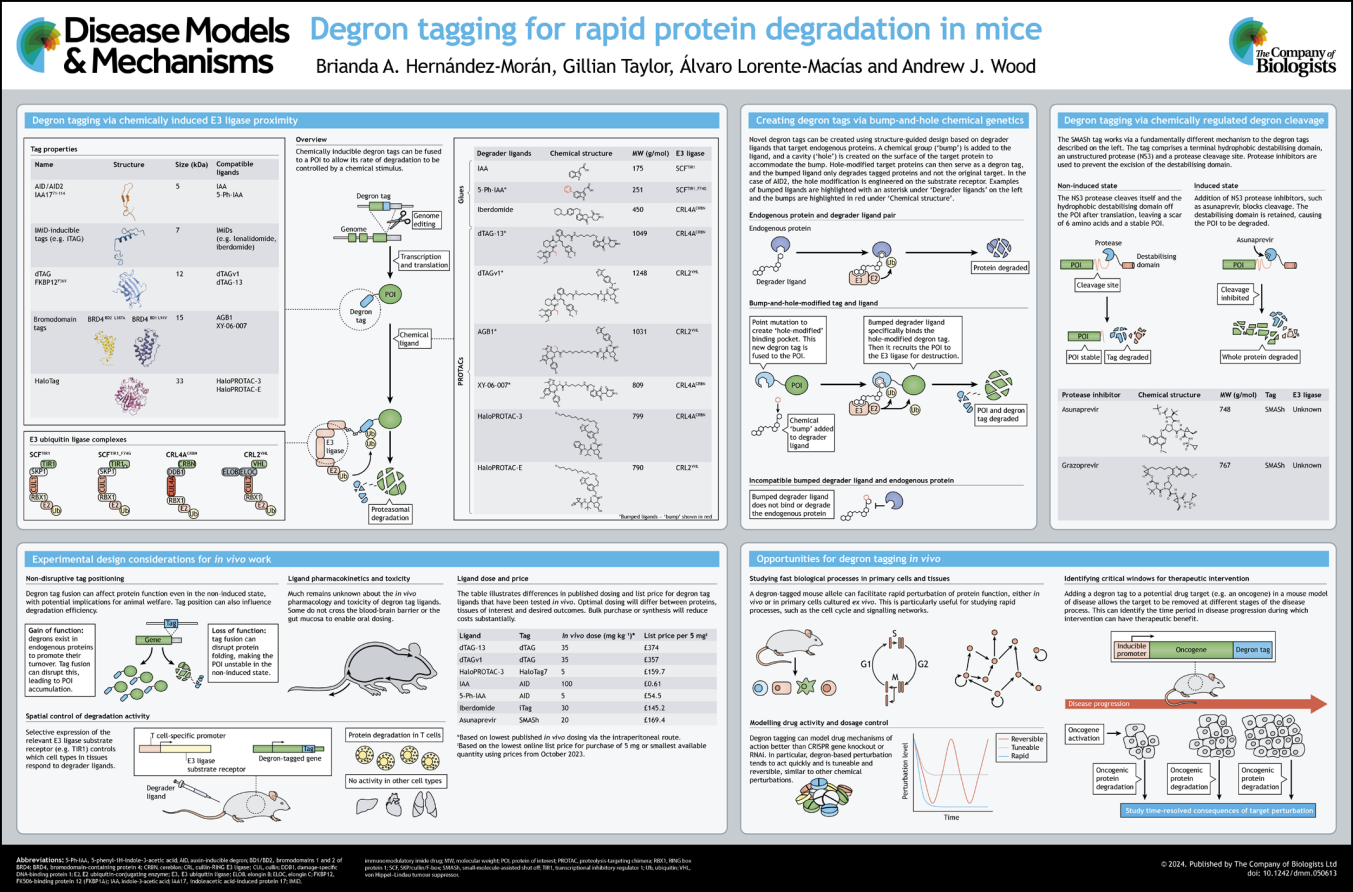
See supplementary information for a high-resolution version of the poster.

## Introduction

The ability to conditionally control gene function has become central to the mouse genetics toolbox in the post-genomic era. Tools such as site-specific recombinases (Box 1), CRISPR nucleases and siRNA have enabled transformative advances in our understanding of healthy development and disease (Chen et al., 2019; Gu et al., 2018; Quadros et al., 2017; Wang et al., 2013). However, each of these tools acts by targeting nucleic acids, whereas most biological functions are carried out by proteins. Nucleic acid perturbations are desirable for some experimental applications, but for others, they can limit our ability to understand human disease mechanisms and model therapeutic interventions:

- **Studying fast biological processes:** After DNA is mutated, protein function is lost gradually, sometimes over several days, owing to the persistence of mRNA and protein produced before the mutation. Removing proteins directly allows mechanistic studies to be performed at acute timescales, making it easier to distinguish the primary effects of protein perturbation from downstream consequences.
  **Box 1. Conditional control of gene function**
  For more than 20 years, the conditional activation of site-specific recombinases has been the standard method for temporal and spatial control of gene function in rodent models. Typically, one or more essential exons of a gene are first flanked with recombinase target sites (e.g. LoxP, FRT) inserted into intronic sequence. Expression of the recombinase (e.g. Cre, Flp) then catalyses recombination between these sites, excising the intervening exonic sequence as a circular DNA fragment and leaving a deletion of the corresponding sequences in the chromosome (Gu et al., 1993, 1994). This system has been adapted to enable different types of genomic rearrangements, including inversions and translocations (Deursen et al., 1995; Oberdoerffer et al., 2003; Zheng et al., 2000), and regulation of recombinase activity can provide temporal and spatial control of gene function (Feil et al., 1997; Gu et al., 1993). For example, fusion of a recombinase cDNA downstream from a tissue-specific promoter will induce recombination, and thus loss of gene function, specifically in cells in which the promoter is active and in their descendants (Gu et al., 1993). Temporal control can be achieved by fusing the recombinase to a mutant form of the mouse estrogen receptor 1 (ESR1) (Schwenk et al., 1998). The fusion protein is cytoplasmic and therefore inactive in the basal state, but shuttles to the nucleus to induce recombination following tamoxifen exposure.
- **Modelling drug activity:** Although DNA mutations are often used to validate drug targets, most drugs work at the protein level. Directly targeting proteins could present a more accurate method to validate potential drug targets in human disease models.
- **Dosage control:** Many human disease states involve changes in protein dosage, which are poorly modelled by manipulating DNA. The ability to titrate protein dosage at different stages of the disease process is therefore highly desirable.
- **Reversibility to identify critical therapeutic windows:** Loss-of-function mutations are often irreversible. Better ways to switch between protein ‘on’ and ‘off’ states in living tissues would enhance our ability to determine whether, and at what critical time points during disease progression, therapeutic interventions could yield clinical benefit.

Over the past decade, genome-editing technologies have greatly accelerated the speed at which genetically engineered mouse models can be generated. One of the next frontiers in mouse genetics is to combine genome-editing capabilities with other technological developments to better serve the needs of researchers engaged in discovery science, disease modelling and drug development. One such advance is the discovery of cell-permeable chemical ligands that promote physical interactions between a target protein [or protein of interest (POI)] and components of the ubiquitin–proteasome system, most frequently an E3 ubiquitin ligase complex (Box 2) (Ito et al., 2010; Krönke et al., 2014; Sakamoto et al., 2001; Tan et al., 2007; Uehara et al., 2017). The resulting ternary complex promotes polyubiquitylation of the target protein and subsequent degradation by the proteasome (Coleman and Crews, 2017). These ligands, hereafter referred to as ‘degraders’, can induce rapid turnover, reducing protein dosage by up to 80-95% within 1-2 h under optimal conditions. Degradation can be tuned to different levels by varying degrader concentrations and can be reversed when the ligand is removed.

**Box 2. E3 ubiquitin ligase complexes**

E3 ubiquitin ligases facilitate transfer of ubiquitin, a 76 amino acid protein, onto specific protein substrates. In many cases, proteins become polyubiquitylated to signal their destruction by the proteasome. E3 ubiquitin ligase complexes are highly diverse, with several hundred assemblies of distinct subunit composition used in mammalian cells in different contexts to degrade a wide range of target proteins (Morreale and Walden, 2016). The most diverse subfamily is the cullin–RING E3 ligases, which comprise a core of four components (see poster, ‘E3 ubiquitin ligase complexes’): a substrate receptor (e.g. VHL, CRBN or TIR1) that directly recognises the target protein, a cullin subunit that serves as a scaffold, adaptor proteins (e.g. SKP1, DDB1 or ELOB–ELOC) that bridge the substrate receptor to the cullin subunit, and a RING finger protein (e.g. RBX1) that recruits an E2 enzyme loaded with ubiquitin.

The specific molecular features on target proteins that are recognised by E3 ubiquitin ligases, and which therefore determine protein half-lives, are termed ‘degrons’. Although some degrons function constitutively, others are regulated by post-translational modifications or via chemical triggers that mediate their interaction with the cognate E3 ligase. Increasingly, chemical biologists are learning how to identify molecules that regulate interactions between E3 ligases and degrons in order to control target protein half-lives. Because degrons function in a modular fashion, it is possible to fuse a degron to a new protein of interest to control the rate of turnover, much like promoter fusions can control spatiotemporal regulation of transcription.

Degrader compounds fall into two main categories: molecular glues and proteolysis-targeting chimeras (PROTACs). Molecular glues, such as auxins [e.g. indole-3-acetic acid (IAA)] and immunomodulatory imide drugs (IMiDs; e.g. thalidomide, iberdomide), are typically monovalent. This means that they preferentially bind to either the POI or the E3 ligase and create a new surface that enables binding with the second protein (Ito et al., 2010; Krönke et al., 2014; Rui et al., 2023; Słabicki et al., 2020; Tan et al., 2007; Uehara et al., 2017). PROTACs consist of two distinct chemical moieties joined by a linker: a ‘warhead’ that engages the target protein and an E3 ligase binder (Békés et al., 2022; Buckley et al., 2015; Sakamoto et al., 2001; Winter et al., 2015; Zengerle et al., 2015). In addition, proximity-inducing molecules are currently being developed to control numerous other cellular processes beyond ubiquitylation (Gourisankar et al., 2023; Henning et al., 2022; Wang et al., 2021). The reader is referred to more detailed reviews on the chemical biology of degrader compounds (Hanzl and Winter, 2020; Hartung et al., 2023; Teng and Gray, 2023) and the expanding scope of chemically induced proximity (Liu and Ciulli, 2023; Stanton et al., 2018).

Huge efforts are currently underway to identify degraders that are effective against proteins of biomedical significance (Békés et al., 2022; Schneider et al., 2021). However, the development of potent and selective degraders for new protein targets remains costly and time consuming, particularly for compounds destined for the clinic, which often require extensive optimisation to achieve desirable pharmacokinetic properties (Pike et al., 2020). An alternative approach leverages both genome editing and protein engineering (see poster, ‘Degron tagging via chemically induced E3 ligase proximity’) to fuse additional peptide sequences to target proteins (hereafter referred to as ‘degron tags’) that interact with E3 ubiquitin ligases in a chemically controllable manner (Bond et al., 2021; Bouguenina et al., 2023; Carbonneau et al., 2021; Nabet et al., 2018, 2020; Nishimura et al., 2009; Nowak et al., 2021; Veits et al., 2021; Yamanaka et al., 2020). This renders the degron-tagged protein susceptible to degradation using generic pre-validated degrader molecules, over time scales of minutes to hours, in a manner that is reversible and dosage controllable.

Degron tags are already widely and successfully used in cultured mammalian cells and invertebrates, in which they provide unprecedented mechanistic insight into fast biological processes, such as gene regulation and the cell cycle (Goldner et al., 2023; Jaeger and Winter, 2021; Wit and Nora, 2023; Wu et al., 2020a; Zhang et al., 2015). In the last few years, several groups have begun to explore their use in genetically engineered mouse models (Abuhashem et al., 2022; Macdonald et al., 2022; Suski et al., 2022; Yenerall et al., 2023; Yesbolatova et al., 2020; Bisia et al., 2023). In this At a Glance article, we begin by briefly covering the chemically inducible degron tag systems that have been used most extensively in cultured mammalian cells. We emphasise the subset that have, at the time of writing, been applied successfully in genetically engineered mouse models. We then provide an overview of the challenges and opportunities with the successful application of degron tagging, focusing on those with greatest relevance to *in vivo* experiments. For a more general overview of protein degradation-based experimental tools, the reader is referred to other recent articles (Chen et al., 2023; Clift et al., 2018; Nabet et al., 2022; Wu et al., 2020a).

## The development of degron tag systems

### Auxin-inducible degrons

Auxins (e.g. IAA) are natural phytohormones that control diverse aspects of plant development and homeostasis (Woodward and Bartel, 2005). They bind to the auxin receptor, transcriptional inhibitory regulator 1 (TIR1), which is an F-box protein that confers substrate specificity to an E3 ubiquitin ligase complex of the SKP/cullin/F-box (SCF) family (Box 2) (Gray et al., 2001; Tan et al., 2007). IAA binding to TIR1 enables the binary complex to recognise a range of auxin-responsive factors (ARFs) via their degron domain (Tan et al., 2007). IAA-inducible docking between TIR1 and ARFs causes their ubiquitylation by SCF^TIR1^, followed by rapid degradation via the proteasome (Gray et al., 2001).

Pioneering work published in 2009 by Masato Kanemaki and co-workers showed that the auxin system could be co-opted as a tool for protein degradation in non-plant cells (Nishimura et al., 2009). Although TIR1 is not found outside the plant kingdom, the remaining subunits of the SCF E3 ligase complex are widely conserved in eukaryotes. Fusing an auxin-inducible degron (AID) peptide derived from an *Arabidopsis thaliana* ARF protein (IAA17) to a target protein is sufficient to enable IAA-responsive degradation of the tagged protein in non-plant cells, provided that a *TIR1* transgene is expressed simultaneously. This degradation occurs via a heterologous E3 ligase complex comprising the plant-derived TIR1 substrate receptor and the endogenous SCF backbone (Nishimura et al., 2009). Subsequent deletion experiments defined a minimal 44 amino acid sequence of IAA17 (IAA17^71-114^) that is sufficient to confer inducible degradation following fusion to the POI (Morawska and Ulrich, 2013).

This system has proven to be effective in a range of species, but has been most extensively used in yeast, *Caenorhabditis elegans* and cultured mammalian cells (Holland et al., 2012; Nishimura and Kanemaki, 2014; Zhang et al., 2015). However, a drawback of this original system was that a subset of degron-tagged proteins was expressed at lower baseline levels even in the absence of exogenous IAA. This ‘leaky’ degradation depended on TIR1 expression (Yesbolatova et al., 2020) and was therefore distinct from the destabilising effects that can occur following fusion of any form of protein tag. The mechanistic basis of leaky degradation and why it affects only some AID-tagged proteins is not clear. However, IAA is a natural by-product of tryptophan metabolism by gut microbes (Lai et al., 2021; Tintelnot et al., 2023) and is present at low levels in mammalian plasma (Masuda et al., 2019), and thus it is conceivable that molecules resembling IAA are introduced via the culture medium.

To address this problem, the Kanemaki (Yesbolatova et al., 2020) and Fukagawa (Nishimura et al., 2020) groups engineered synthetic auxin/TIR1 pairs using bump-and-hole chemical genetics (see poster, ‘Creating degron tags via bump-and-hole chemical genetics’). This method allows new shape-complementary ligand/receptor pairs to be obtained by modifying the binding interface (Baud et al., 2014; Belshaw et al., 1995; Bishop et al., 2000; Clackson et al., 1998; Uchida et al., 2018). A single missense mutation introduced a hole in the auxin-binding pocket of TIR1 (TIR1^F74G^), and a complementary chemical ‘bump’ was added to IAA by attaching a phenyl group at position C5 of the indole ring (5-Ph-IAA) to enable degradation of proteins tagged with the unmodified AID peptide. These alterations resulted in the AID2 system, which reduced the TIR1-dependent basal degradation problem for several tested proteins (Yesbolatova et al., 2020). Importantly, the 5-Ph-IAA ligand is approximately 1000-fold more potent against AID-tagged proteins compared to the original IAA, which is likely to be advantageous *in vivo*.

### IMiD-inducible degrons

IMiDs are phthalimide drugs (e.g. lenalidomide or iberdomide) that function via a molecular glue mechanism. IMiDs bind to the surface of the E3 ligase substrate receptor cereblon (CRBN; Box 2) to induce degradation of new target proteins (Donovan et al., 2018; Krönke et al., 2014; Lu et al., 2013). However, although both mouse and human CRBN proteins bind thalidomide (Ito et al., 2010), a single amino acid change in mouse CRBN proximal to the IMiD-binding site prevents target protein recruitment (Krönke et al., 2015; Petzold et al., 2016).

Several degron tag systems have been developed based on IMiD degrader compounds and their protein targets (Carbonneau et al., 2021; Koduri et al., 2019; Yamanaka et al., 2020). A caveat of these systems was that IMiDs can degrade numerous endogenous (i.e. non-tagged) proteins, making them suboptimal inducers for mechanistic studies in human cells. However, a recent study from Rajesh Chopra and co-workers used a chimeric degron sequence built from motifs found in different human IMiD-dependent degrons (iTAG) that was able to induce rapid degradation of fused target proteins in mouse cells in response to the IMiD compound iberdomide (Bouguenina et al., 2023). Remarkably, the mouse orthologs of human IMiD targets were not significantly degraded. A separate study from the Choudhary, Fischer and Liu laboratories added a chemical bump to an IMiD ligand in order to block the degradation of endogenous substrates (Mercer et al., 2024). A compatible degron tag was then obtained using an elegant directed evolution approach, and this tag/ligand pair could degrade target proteins in both mouse and human cells without off-target activity. At the time of writing, these orthogonal IMiD degron tag systems have not yet been tested in mouse cells *in vivo*. However, potential advantages include relatively small tag sizes (36-60 amino acids) and ligands that resemble clinically approved molecular glue molecules with favourable pharmacokinetic profiles.

### dTAG

The dTAG system, developed by Behnam Nabet, with the laboratories of Nathaniel Gray and Jay Bradner, facilitates rapid degradation of proteins fused to a mutant form of the prolyl isomerase protein FKBP12 (or FKBP1A) (Nabet et al., 2018). dTAG took advantage of PROTAC molecules that had previously been assembled from F506, an immunosuppressive drug that binds to FKBP12, and from IMiD-based CRBN ligands (Winter et al., 2015). To selectively target tagged proteins without affecting endogenous FKBP12, a bump-and-hole approach (see poster, ‘Creating degron tags via bump-and-hole chemical genetics’) was used to engineer the tag/ligand-binding interface. The F36V mutation creates a cavity on the FKBP12 surface that could be engaged by a ligand with a complementary bump modification (dTAG-13) (Clackson et al., 1998; Nabet et al., 2018). The resulting tag/ligand pair enabled degradation of a range of different target proteins with little or no off-target activity (Nabet et al., 2018).

A key feature of PROTACs is the possibility of changing the E3-binding moiety to recruit different catalytic activities to the same target protein. This principle was applied to develop a second PROTAC degrader (dTAGv1) that used the same tag-binding moiety linked to a ligand for the E3 ligase substrate receptor VHL (Box 2), instead of CRBN (Nabet et al., 2020). In this way, different E3 ligase complexes can be recruited to the same tagged protein using distinct degrader compounds. Importantly, dTAGv1 was shown to promote degradation of certain tagged proteins that were poorly degraded by dTAG-13 (Bondeson et al., 2022; Nabet et al., 2020; Olsen et al., 2022).

The potential to use different ligands to reprogram degradation pathways is therefore an advantage of dTAG. In the future, it is possible that additional ligands will become available that enable chemically inducible recruitment of proteins with other biochemical activities to FKBP12^F36V^ fusion proteins. For example, ligands have recently been developed that allow recruitment of proteins with acetyltransferase activity to proteins tagged with FKBP12^F36V^ (Wang et al., 2021).

### Bromodomain tags

Degron tags based on different bromodomains from the BRD4 protein were developed in parallel in the laboratories of Eric Fischer and Alessio Ciulli. These groups used the bump-and-hole approach to engineer bumped PROTAC ligands (XY-06-007 and AGB1, respectively) that selectively target proteins tagged with hole-modified bromodomains to CRBN (Nowak et al., 2021) or VHL (Bond et al., 2021; Runcie et al. 2018), without degrading endogenous BRD4 or off-target proteins. *In vivo* pharmacokinetic data are available for both ligands (Bond et al., 2021; Nowak et al., 2021), but data on their ability to degrade tagged proteins *in vivo* are not yet available.

### HaloTag

HaloTag is a bacterial hydrolase enzyme that ‘self-labels’ by forming covalent linkages to ligands containing a chloroalkane group (Los et al., 2008). By using different chloroalkane-functionalised ligands, this technology has been adapted to multiple applications, including imaging and protein purification (England et al., 2015). Degradation of HaloTagged proteins can be achieved by using HaloPROTAC ligands that recruit tagged proteins to different E3 ligase substrate receptors (Buckley et al., 2015; Tomoshige et al., 2016; Tovell et al., 2019) or by using hydrophobic ligands that cause proteins to be recognised as misfolded (Neklesa et al. 2011). Unlike other degron tag ligands described above, these molecules form covalent linkages with the target protein but not with the E3 ligase subunit (Chen et al., 2022), which cause the HaloPROTAC to be degraded together with the target protein. At 297 amino acids, the tag is substantially larger than either AID or dTAG, but one study reported efficacy for protein degradation in the liver of live mice (BasuRay et al., 2019), and the availability of diverse ligands to recruit new functions to HaloTagged proteins means that a single allele can be used for a range of experimental applications.

### AdPROM

The affinity-directed protein missile (AdPROM) system, developed in the laboratory of Gopal Sapkota, uses a nanobody that binds to a target protein and is genetically fused either directly to an E3 ligase subunit (Fulcher et al., 2016) or, to enable chemical control, to HaloTag (Simpson et al., 2020). In the latter case, HaloPROTACs can subsequently be used to degrade target proteins as described above. Where nanobodies are available against a target POI, AdPROM does not require the target protein to be genetically tagged.

### SMASh

The small-molecule-assisted shutoff (SMASh) system works via a fundamentally different mechanism to the tags detailed above (see poster, ‘Degron tagging via chemically regulated degron cleavage’) and is based on the non-structured 3 (NS3) protease from the hepatitis C virus (Chung et al., 2015; Lin et al., 2008). SMASh tags consist of a short destabilising domain, the NS3 protease and a cognate protease cleavage site. Under basal (i.e. non-induced) conditions, the protease cleaves off the destabilising domain shortly after protein synthesis, leaving a functional target protein with a short peptide ‘scar’ of just 6 amino acids (Chung et al., 2015). Tag cleavage can be inhibited in an inducible manner using small-molecule inhibitors of the NS3 protease, such as asunaprevir. This causes tags to be retained and destabilises newly synthesised target proteins but, importantly, leaves proteins synthesised before inhibitor exposure intact. Because the pre-existing pool is unaffected, the expression of longer-lived proteins can take longer to reduce using SMASh compared to the time taken by other degrons (Bondeson et al., 2022). However, SMASh has proven to be effective on a range of target proteins in cultured cells (Bondeson et al., 2022; Chung et al., 2015; Wu et al., 2020b; Zhu et al., 2019). Furthermore, the fundamentally different mechanism of action could be advantageous for proteins that do not tolerate large tags, or in scenarios in which the retention of previously synthesised target proteins is desired.

## Application of degron tags *in vivo*

In this section, we specifically review the knowledge gained from recent studies using genetically engineered mouse models for the AID, AID2, dTAG and SMASh tag systems. We focus on general properties of these systems for *in vivo* use, rather than insights into the function of specific tagged target proteins.

### AID and AID2

In 2022, two groups including our laboratory and the Sicinski laboratory reported genetically engineered mouse models that enabled endogenously tagged proteins to be degraded using the original AID system (Macdonald et al., 2022; Suski et al., 2022). Intraperitoneal (IP) administration of IAA at 100 mg kg^−1^ was sufficient to degrade 80-95% of two endogenously tagged condensin subunits (NCAPH and NCAPH2) 2 h post injection (Macdonald et al., 2022). Protein levels began to recover within 6 h post injection and returned to baseline within 3 days (Macdonald et al., 2022). In the second study, which used substantially higher doses (up to 800 mg kg^−1^), degradation of the CDC7 kinase persisted for at least 12 h following IP administration and for at least 24 h following oral gavage (Suski et al., 2022). It should be noted that the persistence of degradation activity is determined by both ligand pharmacology and the rate at which the tagged protein is resynthesised once ligand concentrations decline. Degradation was observed in a range of cell types *in vivo* and there were no significant off-target effects on the proteome of thymic T cells following *in vivo* administration (Macdonald et al., 2022). In two other studies unrelated to degron tagging, daily injections of IAA at 50 mg kg^−1^ for 4 (Shen et al., 2022) or 6 (Ji et al., 2019) weeks did not cause weight loss or overt toxicity. However, toxic effects have been reported following much higher doses (500-1000 mg kg^−1^) in mice and rats (Furukawa et al., 2004; Suski et al., 2022).

As discussed earlier, micromolar concentrations of the AID ligand IAA are found in mammalian plasma (Masuda et al., 2019; Lai et al., 2021; Tintelnot et al., 2023). Endogenous levels of IAA were too low to cause TIR1-dependent degradation of tagged proteins in mice, at least for the three AID-tagged proteins tested in germline transgenic models to date (Macdonald et al., 2022; Suski et al., 2022). However, plasma IAA concentrations may vary depending on dietary tryptophan levels and gut microbiota composition (Tintelnot et al., 2023), and it remains possible that other tagged proteins, tested under different experimental conditions *in vivo*, could be susceptible to this problem.

In humans, serum IAA levels have been associated with beneficial and pathological phenotypes. IAA is elevated in the serum of patients with chronic kidney disease (Vanholder et al., 2003), possibly because kidney malfunction reduces the rate at which microbiome-derived IAA is cleared from the body. In other studies, daily IAA administration at 50 mg kg^−1^ was shown to have a protective effect on the development of non-alcoholic fatty liver disease (Ji et al., 2019) and ankylosing spondylitis (Shen et al., 2022). Treatment of mice with 500 mg kg^−1^ of IAA was shown to improve anti-tumour responses to chemotherapy, and serum IAA levels positively correlated with response to chemotherapy in patients with pancreatic cancer (Tintelnot et al., 2023). To summarise these findings, IAA does not appear to have strong acute toxicity at doses in the range suitable for degrading AID tagged proteins *in vivo*. However, IAA is not biologically inert in mammals that do not express TIR1, particularly at higher doses. Further research and careful controls are essential for experiments using this molecule *in vivo*.

In contrast to IAA, the ‘bumped’ AID2 ligand 5-Ph-IAA is synthetic, with no known function in nature, and is substantially more potent (Yesbolatova et al., 2020). In 2020, the AID2 system was reported to work in mouse models, both in xenografted cells engineered to express AID-tagged proteins and TIR1^F74G^ and in tissues from mice expressing an AID-tagged GFP reporter from a randomly integrated transgene (Yesbolatova et al., 2020). Initial studies indicated that >90% of the GFP reporter was degraded in a range of adult tissues following IP injection of the 5-Ph-IAA ligand at 5 mg kg^−1^, and similarly in embryonic tissues following IP injection of pregnant dams. Notably, although less than in other tissues, GFP was degraded by more than 50% in the brain following single-dose IP injection of 5-Ph-IAA (Yesbolatova et al., 2020). The moderate efficacy in brain is likely attributable to incomplete penetrance of 5-Ph-IAA across the blood-brain barrier. No toxic effects were reported following daily administration via the IP route at doses up to 10 mg kg^−1^ over 1 week (Yesbolatova et al., 2020). As discussed above, the AID2 system also alleviated the problem of leaky degradation in cultured cells and in *C. elegans* (Negishi et al., 2021; Yesbolatova et al., 2020). Despite these promising results, more detailed safety studies are needed to exclude subtle phenotypes in response to short-term exposure to 5-Ph-IAA, to better understand the effects of longer-term exposure and to determine whether this molecule or its breakdown products are bioactive in the presence or absence of TIR1^F74G^.

### dTAG

The two commonly used dTAG ligands are PROTAC molecules, which are larger in size and more chemically complex relative to IAA and 5-Ph-IAA. However, it is now well established that effective PROTACs do not adhere to the traditional rules for ‘drug-like’ molecules (Edmondson et al., 2019; Lipinski et al., 2001) and both dTAG ligands can enter cells and degrade tagged proteins when used at nanomolar concentrations in cell culture (Nabet et al., 2020). dTAGv1 and dTAG-13 have an *in vivo* half-life of 4.4 and 2.4 h, respectively, following IP dosing in mice (Nabet et al., 2020).

The dTAG system was first tested in germline transgenic mouse lines by Abuhashem et al. (2022) using alleles with endogenous tags fused to the transcriptional elongation factor NELFB. In preimplantation mouse embryos cultured *ex vivo*, NELFB was profoundly degraded within 1 h of dTAG-13 exposure. Dosing with dTAG-13 or dTAGv1 in pregnant females via IP injection also elicited near complete (>90%) degradation of NELFB in post-implantation embryos 4 h post injection (Abuhashem et al., 2022). This demonstrates that, similar to IAA and 5-Ph-IAA, the dTAG ligands can cross the placenta. A more recent paper reported robust dTAG-mediated degradation of the transcription factor EOMES in pre-implantation embryos *in vitro* (Bisia et al., 2023). However, EOMES degradation was less effective and more variable in post-implantation embryos [embryonic day (E) 5-7] following IP injection of dTAG ligands in pregnant dams, with dTAG-13 providing more complete degradation than dTAGv1. Variability was observed among embryos in the same litter and among cell lineages in individual embryos (Bisia et al., 2023). This likely resulted from restriction of ligand biodistribution *in vivo*, because *in vitro* treatment of stage-matched embryos with dTAG-13 was highly effective.

In adult mice, near-complete degradation of NELFB was evident in several tissues 6 h following IP injection, with the notable exception of the brain, where no degradation was observed (Abuhashem et al., 2022). Intracranial injection of either dTAGv1 or dTAG13 directly into the brain was able to elicit protein degradation, albeit somewhat localised to the site of injection (Abuhashem et al., 2022). This demonstrates that the dTAG system is effective in a wide range of ‘normal’ embryonic and adult cell types, but the dTAG ligands do not traverse the blood-brain barrier.

In a separate study, dTAG fusion alleles were developed for the cyclin-dependent kinase proteins CDK2 and CDK5. In small intestine organoids and bone marrow cells cultured *ex vivo*, over 80% and 95% of protein was degraded, respectively, within 4 h of treatment with 100 nM of dTAG-13, without significant effects on cell viability (Yenerall et al., 2023). Subsequent *in vivo* studies tested a range of pharmaceutical formulations to deliver dTAG-13, which were designed to overcome issues with aqueous solubility of this compound. All were reported to cause some degree of toxicity across multiple tissues after daily dosing up to 26 days, but this was primarily attributed to the formulation rather than the compound itself because toxicity was observed even in mice treated with vehicle only (Yenerall et al., 2023). A more toxic formulation (ethanol, PEG400, Tween 80; 20:60:20) allowed subcutaneous delivery of dTAG-13 at up to 300 mg kg^−1^, which produced robust protein degradation across most adult tissues at 4 h following sub-cutaneous injection. Administration using a less toxic formulation, which allowed delivery at 15 mg kg^−1^, produced relatively modest effects on target protein levels (<50% degradation in most tissues) (Yenerall et al., 2023).

These findings highlight the complex interplay between effective dose, compound solubility and vehicle toxicity, which must be considered for *in vivo* studies using compounds with hydrophobic properties. However, we note that other studies have achieved more robust degradation of dTAG fusion proteins following IP injection of dTAG-13 at 35 mg kg^−1^ in different formulations (Abuhashem et al., 2022; Nabet et al., 2018, 2020). Moreover, the dTAGv1 ligand, which was not analysed in the CDK2/CDK5 study, has pharmacokinetic properties that could be more favourable for *in vivo* experiments in mice (Nabet et al., 2020). This ligand substantially degraded a luciferase reporter protein, with activity persisting for up to 28 h following injection at 35 mg kg^−1^ in xenografted tumour cells (Nabet et al., 2020). Moving forward, detailed studies looking at multi-tissue toxicity following repeat dosing regimens of dTAGv1 would be of value.

### SMASh

The degradation of SMASh-tagged proteins is induced using the protease inhibitor asunaprevir, which has been approved for clinical use in humans (Akamatsu et al., 2015). Detailed pharmacokinetic and toxicology data are therefore available, which support the potential for safe use, including oral dosing, in mouse models (McPhee et al., 2012). However, asunaprevir does not traverse the blood-brain barrier (McPhee et al., 2012), potentially limiting the use of this system for conditional protein degradation in the brain.

The first reported use of the SMASh tag system in a genetically engineered mouse model involved fusion of SMASh, together with an mCherry reporter, to the C-terminus of the PD-1 (PDCD1) protein, a cell surface receptor involved in immune checkpoint control and an important target for cancer immunotherapy (Naruse et al., 2022). However, the tag was found to reduce the level of PD-1 even in the absence of protease inhibitors, and mice developed glomerulonephritis and arthritis upon ageing, likely owing to partial loss of PD-1 function in the non-induced state. Of note, the allele design in this study meant that the mCherry reporter remained fused to PD-1 following cleavage of the SMASh tag in the non-induced state, which may have contributed to reduced PD-1 expression and function. This highlights the importance of careful tag site selection during allele design, which is discussed further below. Nonetheless, addition of protease inhibitors reduced the levels of PD-1 protein further, to a degree that was sufficient to promote tumour killing in a syngeneic tumour model (Naruse et al., 2022).

## Experimental design considerations for *in vivo* work

As outlined above, degron tagging systems developed in cell culture have, based on the limited number of studies thus far, shown promising results in genetically engineered mice. In this section, we outline the experimental design considerations that are of particular importance when designing a strategy for degron tagging *in vivo*, which could help to improve the chance of successful outcomes (see poster, ‘Experimental design considerations for *in vivo* work’).

### Tag positioning

The fusion of peptide tags to target proteins is essential for degron-mediated protein degradation, as well as many other applications in molecular biology. However, the design of degron tagged proteins is constrained by two requirements.

Firstly, the tag should be fused in a way that does not significantly interfere with the normal expression or function of the POI. Poorly positioned tags can disrupt protein folding to reduce stability in the non-induced state or change localisation, dynamics or intermolecular interactions (Crivat and Taraska, 2012). Tagging at the N- or C-terminus can be effective in many cases, but, in others, the target protein is perturbed to a degree that is no longer useful (Bendezú et al., 2009, 2015). In these cases, placing tags at internal sites corresponding to flexible loop regions could prove effective.

Appending a tag to any protein is likely to alter function to some degree. Although minimal perturbation is typically the goal during allele design, moderate tag effects might be tolerable for ‘quick and dirty’ overexpression experiments in cancer cell lines. Tagging endogenous genes via genome editing in the mouse germline is a more sensitive scenario in which small perturbations can have large effects on animal development and welfare. Minimally disruptive tagging is therefore important for the ‘3Rs’ principles of ‘reduction’, by reducing the chances of failed animal experiments, and ‘refinement’, by minimising harms resulting from genetic modification. For these reasons, there is an obligation to test the consequences of tags on normal protein expression and function in non-animal models before proceeding with *in vivo* work.

Secondly, the tag should be fused in a way that enables the POI to dock with the relevant E3 ligase complex, in a drug-inducible manner that facilitates transfer of ubiquitin onto surface lysine residues of the target protein. Although most degron-tagged proteins that are stably expressed can be degraded to some extent, recent evidence suggests that N- and C-terminal degron fusions on the same POI can have distinct efficiencies (Bondeson et al., 2022). Although the reasons for this are unclear, changing the tag position will orient the target protein in a distinct configuration within the ternary complex, which could influence the efficiency of ubiquitin transfer and subsequent degradation (Bondeson et al., 2022; Donovan et al., 2020). In instances in which target protein degradation is suboptimal in cell culture experiments, it would therefore be prudent to evaluate different fusion sites and tags before proceeding with *in vivo* experimentation (Bondeson et al., 2022). Employing different PROTAC ligands for the recruitment of distinct E3 ligases to the same tagged protein (e.g. dTAG-13 or dTAGv1 ligands), can also improve degradation in some circumstances (Nabet et al., 2020).

### Ligand pharmacokinetics

In cell culture, a single dose of degrader compound can elicit durable effects on protein degradation and rapidly achieves uniform distribution. The behaviour of degrader compounds following *in vivo* administration is inherently more complicated. Although high drug concentrations can often be achieved immediately following single-dose administration (Nabet et al., 2020; Yenerall et al., 2023; Yesbolatova et al., 2020), the length of time that ligands persist in the circulation at concentrations that promote degradation of target proteins is affected by the rate at which compounds are metabolised by the liver and cleared via the kidneys. Moreover, after entering the circulation, a large fraction of the drug is typically sequestered via binding to serum proteins, and the size of this protein-bound fraction influences potency, biodistribution and the rate of clearance.

Another important consideration for *in vivo* work is the presence of structures within several mammalian tissues that act as a barrier to xenobiotic substances, preventing their accumulation in certain areas of the body. Examples include the gastro-intestinal epithelium (Bohets et al. 2001), blood-brain barrier (Pardridge, 2012), placenta (Eshkoli et al., 2011) and Sertoli cell barrier within the testes (Meng et al., 2022). In general, drugs with lower molecular mass and higher lipophilicity are better able to traverse membranes compared to larger, hydrophilic compounds (Lipinski et al., 2001). Consistent with this, the molecular glue compound 5-Ph-IAA was able to achieve at least moderate levels of protein degradation in the brain following IP injection (Yesbolatova et al., 2020), whereas the much larger PROTAC ligands dTAGv1 and dTAG-13 were not (Abuhashem et al., 2022; Yenerall et al., 2023). Similarly, protein degradation activity has been observed following oral administration of IAA (Suski et al., 2022), potentially avoiding the need for repeated injections in longitudinal studies. Achieving orally bioavailable PROTAC molecules is challenging, though not impossible (Kofink et al., 2022; Poongavanam and Kihlberg, 2021), but data on oral dosing of dTAG ligands have not been published.

### Tissue-specific E3 ligase activity and spatial control of degradation activity

Although there is current interest in developing ligands against diverse E3 ligases, most PROTACs that have been developed, including those that target degron tags (Bond et al., 2021; Nabet et al., 2018, 2020; Nowak et al., 2021; Veits et al., 2021), recruit the cullin–RING E3 ligase (CRL) complexes CRL2^VHL^ and CRL4A^CRBN^. This has been driven by ligand availability, the broad expression of these complexes across cell types (Uhlén et al., 2015) and their ability to degrade diverse neo-substrates. Nonetheless, it remains possible that context-dependent expression of these substrate receptors or core components of their CRL backbone could have a bearing on the dose response and/or durability of protein degradation in some cell types. In the case of SCF^TIR1^, the complex used by the AID system, it was shown that erythroblasts were not competent for IAA-inducible degradation (Macdonald et al., 2022). Erythroblasts expressed the transgene-derived TIR1 subunit at levels comparable to those in IAA-responsive cell types, raising the possibility that one or more endogenous components of the ubiquitin–proteasome system required for IAA-inducible degradation was lacking in this cell type (Macdonald et al., 2022).

In recent decades, it has been possible to achieve tissue-restricted gene knockouts in mice by first introducing recombinase target sites on either side of one or more essential exons in the gene of interest, and then driving the expression of the corresponding recombinase protein from a tissue-specific promoter (Box 1) (Tian and Zhou, 2021). In *C. elegans*, a similar approach was shown to enable tissue-restricted protein degradation activity following systemic administration of IAA or 5-Ph-IAA (Hills-Muckey et al., 2021; Zhang et al., 2015). When TIR1 is expressed in a tissue-specific manner, the IAA-responsive SCF^TIR1^ complex should only form in a specific subset of cells. To enable this spatial control, the *Rosa26^TIR1^* transgene used by Macdonald et al. (2022) was originally generated with a Lox:Stop:Lox sequence upstream from the *TIR1* open reading frame. Although yet to be demonstrated in mice, this potentially allows spatial expression to be controlled using one of the many Cre driver lines available within the mouse genetics community. The SCF^TIR1^ complex will then form and potentiate a response to IAA only in Cre-expressing cells, restricting the activity of the systemically administered ligand to those sites.

Engineering cell type-specificity into PROTAC-based degradation systems is a major focus of current human drug development efforts. For example, if target proteins can be degraded via recruitment to E3 ligases with higher activity in cancer cells versus that in normal tissue, this could minimise the side effects of PROTAC-based therapeutics in healthy tissues. Although currently available ligands for the dTAG system harness E3 ligases with broad expression (Uhlén et al., 2015), it is conceivable that new PROTAC tool compounds could enable tagged proteins to be degraded via E3 ligases with more tissue-restricted activity (Hoegenauer et al., 2023).

### Financial considerations

The structural complexity of PROTAC ligands used for the dTAG systems makes them time consuming to synthesise, particularly at scale. This is reflected in the relatively high price charged by commercial suppliers for these compounds compared to the less complex ligands for the AID/AID2 and SMASh systems. In cell culture-based experiments, relatively small culture volumes can be used to minimise ligand usage; however, quantities required for a typical *in vivo* experiment are calculated based on milligrams of drug per kilogram of body mass (mg kg^−1^), and thus determined by organism size. The poster (see ‘Ligand dose and price’) provides details of commercial ligand pricing for small batch purchase and published dosing for IP administration (BasuRay et al., 2019; Bouguenina et al., 2023; Macdonald et al., 2022; Nabet et al., 2018, 2020; Naruse et al., 2022; Yesbolatova et al., 2020). However, owing to metabolism and clearance, *in vivo* experiments requiring sustained degradation activity will require repeat dosing. For experiments involving cohorts of animals, dosed over periods of weeks or more, it is important to consider ligand costs at an early stage of project planning.

## Future prospects and conclusions

Although the challenges outlined above are significant, if they can be successfully navigated then degron tagging offers a range of new experimental possibilities to better understand how specific proteins function in normal physiology and disease and to more accurately predict their value as therapeutic targets (see poster, ‘Opportunities for degron tagging *in vivo*’). In particular, rapid removal of proteins facilitates the investigation of dynamic cell signalling networks. Furthermore, degron-tagging may provide a better way to model drug activity by tuning protein dose rather than complete loss of protein function, and the reversibility of degron technology could allow researchers to identify temporal requirements for disease drivers during disease progression and establish critical windows for therapeutic intervention.

In the short term, more clearly defined pharmacokinetic and toxicology profiles are needed for the tool compounds that are currently available to degrade tagged proteins, both to determine their suitability for long-term studies and to ensure that minimally invasive procedures are used for delivery. It is not yet straightforward to degrade secreted proteins or membrane proteins that lack taggable cytosolic or nuclear domains. Emerging strategies that take advantage of membrane-localised E3 ligases (Cotton et al., 2021; Marei et al., 2022) or different cellular degradation pathways, such as the endosome/lysosome system (Banik et al., 2020; Ji et al., 2022; Zhang et al., 2023 preprint), are being adapted to address this problem.

Given the level of investment required to generate genetically engineered mouse models, the field would also benefit from new chemical tools to diversify the potential use of a single allele for different experimental purposes. For example, libraries of tag-compatible degrader ligands with diverse pharmacokinetic properties *in vivo* would help to define the optimal parameters for clinical drug candidates in target validation studies. PROTAC molecules that enable proteins with distinct functional activities (e.g. fluorophores or other post-translational modifiers) to be recruited to the same tagged protein, as exemplified by the HaloTag (Chen et al., 2022; England et al., 2015) and dTAG (Nabet et al., 2018, 2020; Wang et al., 2021) systems, will enable a single tagged allele to be used for a range of applications.

Targeted protein degradation is an area of intense research in both academic and industrial settings, and further advances in the coming years will continue to benefit researchers working with mice and other model organisms. Our vision is that degron technologies will complement, extend and, in many cases, replace recombinase-mediated conditional alleles, simultaneously reducing and refining animal use while producing data that are easier to interpret.

## Poster

Poster

## High-resolution poster

A high-resolution version of the poster is available for downloading at https://journals.biologists.com/dmm/article-lookup/doi/10.1242/dmm.050613#supplementary-data.
