# Supplementary figures and images for: Degron tagging for rapid protein degradation in mice
Source: Dis Model Mech. 2024 Apr 26;17(4):dmm050613. doi: 10.1242/dmm.050613 (PMC11073515; doi:10.1242/dmm.050613)

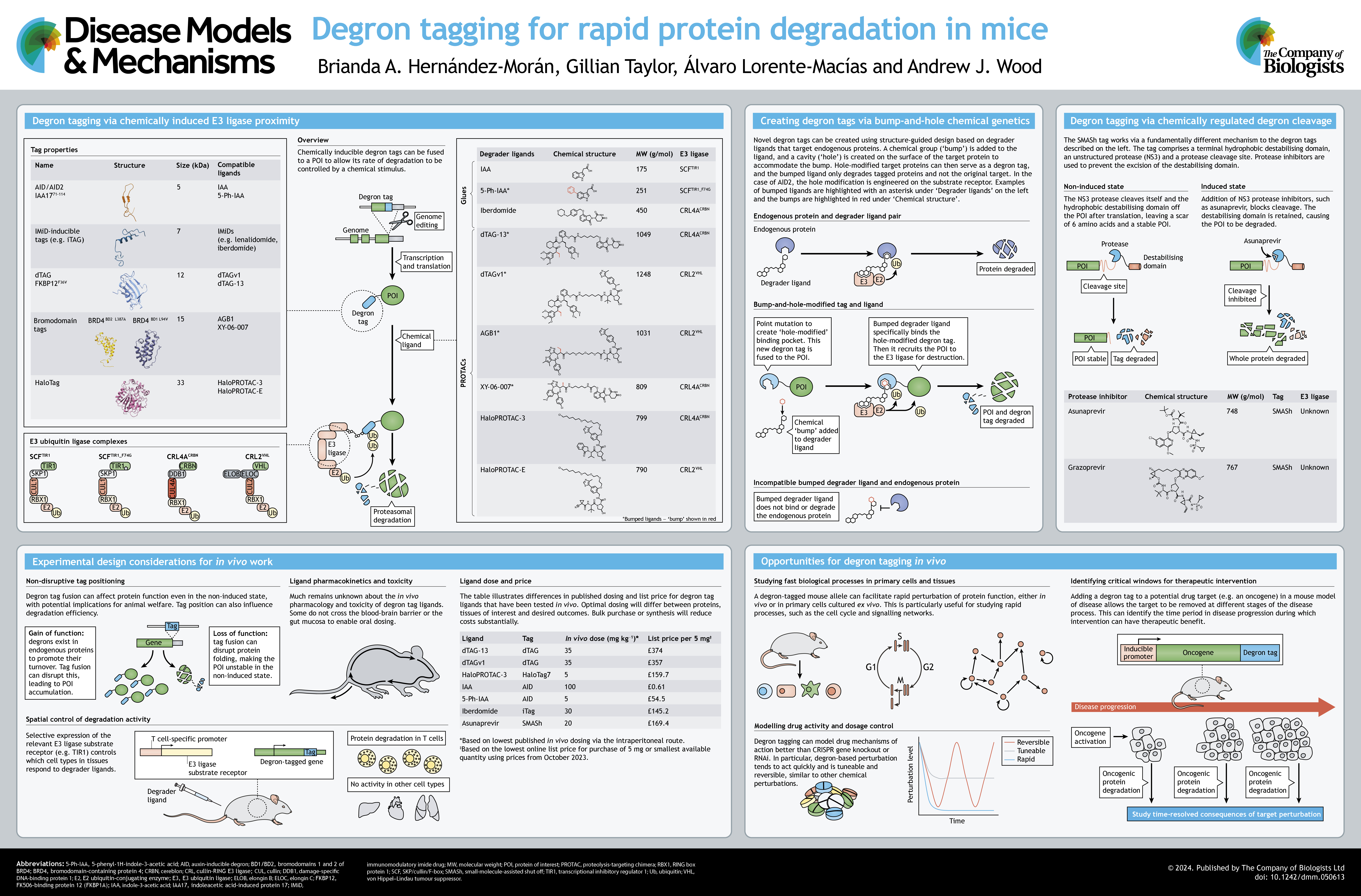

Supplement: Poster [file dmm-17-050613-s1.jpg]
